# Local Anesthesia with Eucain and Eucain-Adrenalin

**Published:** 1904-10

**Authors:** 


### Local Anesthesia with Eucain and Eucain-Adrenalin.

Otto Simon, First Assistant in Prof. Czerny's Surgical Clinic at Heidelberg, (*Muench. Med. Wochenschr.*, July 19, 1904,) says: the number of operations at Czerny's clinic and dispensary increased respectively from 1,717 and 1,035 in 1897, to 1,955 and 1,502 in 1902. The increase in the number done by local anesthesia alone was from 91 and 21 in 1897, to 185 and 193 in 1902. During the latter year he witnessed the death of a patient from cocain anesthesia. Only 7 c.c. of a 1-per-cent. cocain solution had been injected into the urethra, but convulsions, arrest of the heart and respiration followed immediately. The patient was a young man with sexual neurasthenia and chronic prostatitis. The fluid had remained in the urethra only two minutes at the utmost.

Since then eucain has been exclusively used for anesthesia, with or without adrenalin, and it has been found perfectly satisfactory. It is comparatively nontoxic, and an isosmotic; warmed solution induces anesthesia as effectively as cocain in the same strength. Adrenalin enhances its action, and concentrations of 1 to 20,000 are free from by-effects in subcutaneous injections. The Oberst technic is preferred; 188 operations done with eucain are given. The supplementary adrenalin was particularly valuable in extirpation of deep tumors, lipomata, adenomata of mamma and struma, excision of small tumors of tongue and lips, angiomata, in operating on the jaws, and in small plastic operations. Eucain is particularly useful as a preliminary to cystoscopy.—*Journal of the American Med. Asso.*, August 27, 1904.

